# Process and Dynamics in AI and Language Use

**DOI:** 10.1111/tops.12789

**Published:** 2025-02-11

**Authors:** Eleni Gregoromichelaki, Gregory J. Mills

**Affiliations:** ^1^ Department of Philosophy, Linguistics, and Theory of Science University of Gothenburg; ^2^ Department of Computing, Goldsmiths University of London

**Keywords:** AI and language, DS‐TTR, Interactivist model, Bickhard, Process metaphysics, Symbol grounding, Peircian semiotics, Ecological Psychology, Dynamical hypothesis

## Abstract

In this volumed, Randall Beer and Joanna Rączaszek‐Leonardi have opened an important discussion of what is further needed to enhance the reach of dynamical approaches to cognition. Focusing on issues concerning the nature of language and developments in language technology, we have attempted, in this brief contribution, to place their proposals in a larger philosophical framework that suggests lines of inquiry that we believe will yield fruitful outcomes. In particular, we suggest that the adoption of a process metaphysics suggests that dynamic approaches appropriately conceived within the context of current scientific advances are at basis adequate as a framework; however, the more profound implications of its adoption have not yet been sufficiently explored.

## Abstract 2

In this commentary, we focus on issues concerning the nature of language and developments in language technology suggesting that while the adoption of a process metaphysics provides a sound foundation for dynamic approaches, the more profound implications of its adoption have not yet been fully explored.

## 1 Introduction

Until recently, a prevalent view in cognitive science and philosophy has been the employment of internalistic and static models following the symbol‐processing computer metaphor account of the mind/brain (see, e.g., Kelty‐Stephen et al., 2022). Theoretical and formal/computational accounts of language have followed along the same lines since it has seemed obvious that language is the par‐excellence symbolic system. This view of mind and language has led to various questions in science and technology regarding its inherent limitations. One such question in Artificial Intelligence (AI) and Natural Language Processing (NLP) is *the symbol grounding problem*, namely, how it is possible, or whether it is necessary, for formal material/energetic objects (symbols) processed by computational systems to be linked to the external world (see, e.g., Harnad, 2024). We believe that relative to such questions, recent advances in AI and NLP point to the need to abandon the static/internalist perspective on complex functional systems like artificial, social, and biological agents as entities who operate by manipulating internal objects characterized as symbols. Instead, we need models of the phenomenal experience of an ever‐changing unfolding of cognitive activity and, more generally, the dynamicity of physical processes in the environment to which agentic systems are coupled (see, e.g., Noë, 2004; Bickhard, 2009). The static/internalist perspective is at odds with current theories of how agent−world interactions should be interpreted as *constituting* both mind/cognition and the sense of an individuated self (see, e.g., Hutchins, 1995; Kyselo, 2014), as opposed to the standard view of representations and computations controlling the sociomaterial environment by replicating its structure agent‐internally.

Randall Beer and Joanna Rączaszek‐Leonardi are pioneers in articulating the dynamical perspective and formulating models that have contributed profound insights to the analysis of cognitive systems. Therefore, it is highly instructive and illuminating to see how they view the current state of developments in their respective fields. In this volume, Beer and Rączaszek‐Leonardi consider the status of the dynamical perspective for understanding and modeling complex adaptive systems like human behavior, including language. In this commentary, we will focus on the nature of language and interaction and the implications of this view on characterizing the progress that might be possible in AI.

## 2 Dynamics in context

Beer, while endorsing dynamical methodologies, nevertheless, raises doubt about the appropriateness of the *ontological* hypothesis that cognitive systems *are* dynamical systems. If not restricted to the details of the formulation of this hypothesis in van Gelder's (1998) seminal article, this might be taken as recommending the adoption of an instrumentalist perspective on the value and results of dynamical analyses. However, this stance would seem insufficiently ambitious given current evidence for a fundamental process metaphysics across disciplines. Standard “substance metaphysics” takes reality as consisting of static entities and their relations. In contrast, a metaphysics where processes are the fundamental ontological elements takes change as basic and stability as needing explanation (Seibt, 2024) with quantum fields being the current most basic level of material explanation.

Dynamical modeling does not necessitate a process metaphysics (Bickhard, 2020). However, at least in our view, it is the other way round: process metaphysics requires fundamentally dynamic models of cognition, life, and nature. For example, it is becoming widely accepted that the transition from inorganic processes to life and cognition can be modeled as the gradual differentiation of self‐organizing activity patterns out of the interaction of primordial substrate processes that eventually leads to biological and sociocultural activities (see, e.g., Bickhard, 2009, 2024; Seibt, 2018; Deacon, 2012; Kauffman, 1993). This processual perspective is supported by recent interpretations of advances in physics (e.g., Cahill, 2005), relational interpretations of quantum mechanics (Laudisa & Rovelli, 2021), biology (e.g., Dupre & Nicholson, 2018), and category‐theoretic results in mathematics, which can be applied to the formalization of specific ideas about the nature of cognitive phenomena like meaning in language (e.g., the Yoneda lemma, Bradley et al., 2022; action opportunities, *affordances*, Hirota et al., 2024). Life‐ and mind‐related phenomena like normativity, function, and historicity are then emergent properties of recursive dynamic process interactions, with downward causation supplementing the bottom‐up causality available in standard reductionist models. In contrast, dynamics under a simplistic construal that only refers to classical Newtonian physical laws has only recourse to Cartesian dualism or computational functionalism to explain such phenomena.

Emergence and downward causation have been treated with suspicion in frameworks grounded in substance metaphysics while they are natural properties of process models. From this perspective, the methodological suggestions and conceptual considerations advocated by Beer (2023) who recommends models embracing the incremental progression from basal cognition to sophisticated cognitive behaviors (the “continuity principle”) are a natural consequence of a dynamic processual ontology. We believe though that an even more radical stance is required to acknowledge the material/energetic constitution of both agents and their interaction with sign processes because embracing emergence and the nondeterminism of modern physics indicates abandonment of human exceptionalism in favor of recognizing both biological and nonbiological forms of agency (Barad, 2007; Haraway, 2008; Haraway & Goodeve, 2018). Issues like the apparent intractability of the grounding problem are symptoms of neglecting these aspects of human embeddedness. We turn to examine this problem next as an instance of the conundrums that ensue when viewing human cognition as separable and distinct from other forms of mind in nature.

## 3 Deflating the grounding problem

The earlier version of the grounding problem can be addressed through the account of Rączaszek‐Leonardi (2009, 2012, 2016), who combined Ecological Psychology's (EP, see, e.g., Segundo‐Ortin & Raja, 2024) action focus and the dynamical perspective to develop an illuminating account of linguistic “symbols,” as constraints on the “social physics” of interaction. This reconceptualization of the notion of “symbol” as a function defined within coordinative structures that underpin social actions makes it compatible with EP. This development is highly significant and well‐motivated as it falls within other recent attempts to extend the reach of EP to domains standardly thought to be outside its immediate concerns or its toolbox, for example, fields of study like neuroscience (e.g., Favela, 2023; Raja, 2018) and sociocultural affordances (e.g., Mangalam et al., 2024). Rączaszek‐Leonardi's notion of symbols is not the standard code view of decontextualized forms that need to be “grounded” through some type of referential semantics defining correspondences with objects in the world. Instead, they are particular types of affordances, enabling or inhibitory constraints on the dynamics (see also Juarrero, 2002, 2023).

The crucial point for us is that this explication of “symbolic function” focuses on the relatively stable material/energetic constitution of such forms, which underwrites their replicability and historicity within a flux of processes. We can then explain the function of language forms as interaction of multiscale dynamics: the slower dynamics of established structures allows them to act as catalysts (experienced as “meanings” or functions) in the environment to canalize the fast‐scale dynamics that constitute human social interaction. However, one should note here that linguistic forms are not fully stable and replicable actions, especially in verbal communication, as they do change and adapt modulated by other features of the context. Therefore, these forms cannot be defined independently of their participation in networks of interacting elements as the coordinative structures that differentiate and define them. Like other types of memory/information structures, their utility lies in being able to provide salient affordances in the circumstances of use rather than fidelity of form/content reproduction (see also Levin, 2024). Written forms are less evanescent in this respect and have wider reach so that they can more independently constrain dynamics of larger organizations of linguistic behaviors like narrative structures, genres, tone, and styles across larger timescales while complementing other semiotic resources, for example, images in multimodal datasets that underpin current AI technology. This organizational capacity of textual forms is what allows Large Language Models (LLMs) to achieve successful performance in capturing the large‐scale patterns of contextualized behavior of such forms across huge datasets constrained by users’ textual prompts and prior feedback‐based training by humans (RLHF, Ziegler et al., 2019). During learning, the predictive training regime utilized by LLMs in combination with the “attention” search mechanism for capturing superordinate recursive long‐distance relations enables them to capture affordances for continuation of the current text due to constraints that have been extracted from recursively organized regularities in large amounts of training data.

### 3.1 The symbol‐ungrounding model

In the fast dynamics of human verbal behavior, symbolic function as constraint is an instance of what Beer (2023) characterizes as the continual adaptation of the entire agent−social‐environment system under the influence of only partial attractors since coordination, adaptation, and learning are not distinguishable from behavior. This view of symbols thus integrates so‐called linguistic “symbols” with other interactional routines and nonverbal behaviors, which can equally well be explicated as stabilizing and enabling constraints in the social dynamics of interaction. We have argued that all these are constantly under revision and can develop spontaneously and idiosyncratically even during single instances of interaction (Mills and Gregoromichelaki, 2010; Mills, 2014; Healey et al., 2018).

Approaches like Rączaszek‐Leonardi's as well as other dynamic, embodied, and enactive approaches to human cognition and AI (see, e.g., Froese & Taguchi, 2019) have transformed the original version of the symbol grounding problem. In a reversal of the original question, Rączaszek‐Leonardi (2025) formulates a challenge that neither computationalism nor the dynamics‐focused perspective can adequately explain how ability to use language symbolically emerges from and is supported by ongoing coupling with the sociomaterial environment: “How can any form used by a living cognitive system become partly *ungrounded* (Rączaszek‐Leonardi & Deacon, 2018) from the ongoing stream of events to be amenable to rule‐based manipulation …?”

With this challenge as background, Rączaszek‐Leonardi et al. (2018) have updated the earlier view of symbols‐as‐constraints to describe symbol “ungrounding,” that is, the stage in infant development when constraints in interaction can be fulfilled by relations between linguistic forms rather than those forms constraining the interactional dynamics directly. This idea is substantiated by a more general notion of constraints developed by Deacon (2012) along with a Peircian gloss to accommodate the presumed inadequacy of the dynamics to provide grounds for intentionality, function, and normativity. We believe that Deacon's Peircian gloss on a set of otherwise plausible assumptions has undesirable consequences in that the grounding problem resurfaces as an unresolved conundrum given the reformulation, rather than rejection, of the premises that gave rise to it in the first place. We turn to this issue next.

### 3.2 Deacon's Peircian model of constraints

Deacon, under insightful processual ontological assumptions, proposes a reconstruction of traditional notions inherited from computational/symbolic approaches like symbol, meaning, value, reference, and information as *absential* phenomena engendered through interactions of constraints in a multilevel architecture (see also Deacon, 2021). The account of symbolic function relies on an interpretation of Peircean semiotics first developed by Deacon (1997). Accordingly, the process of “ungrounding” invokes a hierarchy of interpretation processes related to a typology of signs based on the Peircian icon‐index‐symbol taxonomy. In a scheme reminiscent of a “picture theory of meaning” (Wittgenstein, 1961), the highest level of this hierarchy, the set of differentiated “linguistic symbols,” is taken to gradually emerge in language learning and coming to constitute an independent systemic layer that needs to be associated with a referential layer and a set of iconic/indexical relations among signs to remain grounded (Rączaszek‐Leonardi et al., 2018: Fig. 6).

Despite its affinity with the process metaphysics advocated here, in our view, certain key phenomena are not easy to reconcile with the semiotic component of the symbol‐ungrounding framework. First, for a model of absential phenomena to do the job of revealing what exactly the effects of the constraints are, an underlying model of the types of modal potentialities that are prevented from being realized while at the same time influencing the dynamics needs to be provided. Otherwise, the constraints cannot be interpreted as ecological information, that is, opportunities for action or *enabling* constraints. Models that describe such influences are anticipatory rather than correspondence‐based (Bickhard, 2009, 2024). Within the framework of DS‐TTR (*Dynamic Syntax with Type‐Theory‐with‐Records*, Gregoromichelaki et al., 2022; Purver et al., 2010, 2011; Hough, 2015; Gregoromichelaki, 2018), words, morphology, syntax, and ambient conceptual structure are all modeled as indicators of opportunities for (inter‐)action. These are expressed in terms of conditional and goal‐driven actions whose accomplishment either gives rise to anticipations of further actions or leads to abandonment of the current strategy due to its nonviability in view of more competitive alternatives (Gregoromichelaki, 2018; Gregoromichelaki et al., 2019, 2020a,b). Participants’ opportunities for action and their perspectives are articulated in a unified model of the whole distributed system (see also Cowley, 2009; Steffensen & Cowley, 2021) that stands for the *landscape of affordances* (Rietveld et al., 2018; Bruineberg et al., 2019; Bruineberg & Rietveld, 2014, 2019). Hence, the claim that grammars should be seen as comprising a set of skills for navigating interaction affordances under the guidance of social practices rather than language‐world correspondences or structure/form versus meaning/function distinctions.

An account in a similar spirit is given in Rączaszek‐Leonardi et al. (2022) with thoughtful and enlightening descriptions of data from language development. However, from our perspective, the Peircian interpretation of the framework appears to be inconsistent with the requisite explanations. The general reason, in our view, is the two trichotomies that are operative in a Peircian account. First, the icon‐index‐symbol typology; second, the decomposition of sign use into relations among sign‐vehicle (*representamen*), *object*, and *interpretant*. We will briefly consider the first of those here and the second in the next section.

Regarding the first trichotomy, we believe that it does not sit well with the original view of symbols as constraints. The priority assigned to iconic relations by Deacon does not seem justified in general, and, from an EP perspective, it seems to prioritize classification instead of affordance perception. Arguably, the notions included under “indexical” relations are just as fundamental if not more in perception/action, especially under a process ontology with anticipation as the main organizing principle. Anticipation can be taken (at a stretch) as indexical processing, which is what grounds implicit notions of representation in processual models (Bickhard, 2024). Moreover, from an empirical point of view, human interaction does not provide any instances of pure iconism or indexicality that can then be used as the basis for symbol ungrounding. All examples mentioned by Rączaszek‐Leonardi et al. (2018) and (2022) as cases of iconic/indexical relations grounding symbols involve, in fact, mixed iconic/indexical and symbolic capacities under any Peircian interpretation, including Deacon's.

We now turn to the second trichotomy defined in the Peircian model.

### 3.3 Language use, current AI, and form versus function

#### 3.3.1 Current AI and grounding of forms

In fact, we believe that the need to view iconism as primary and a potentially independent aspect of reified sign vehicles arises from taking a traditional linguistic or anthropocentric view of semiosis as a triadic phenomenon combined with remnants of a correspondence theory of sign use in Deacon (1997, 2012). In establishing a distinction between sign vehicle and its corresponding object, the framework maintains a traditional distinction between form/matter and meaning/function, which then leads to the need for an independent interpreter as an external observer to resolve this differentiation. In this respect, ultimately the model does not provide an appropriate ontology for conceptualizing the continuity between human and nonhuman cognitive and agential capacities. We will illustrate this difficulty with a debate currently taking place in the domain of AI (for a more general perspective see, e.g., Gamble et al., 2019).

Current LLMs (e.g., GPT‐4) display impressive capacities in terms of producing fluent immediate responses with coherence to the questions posed across a variety of language‐related tasks while being able to adapt to various persona/tone/style/genre as requested by the user. Additionally, multimodal systems are now available that can follow verbal instructions to create images and video as well as display some minimal interaction with the environment (e.g., GPT‐4o). These models are trained by processing disembodied textual and image data and, in recent systems, audio and video. Their impressive performance has been argued to refute the Chomskyan paradigm of language acquisition (Piantadosi, 2024) and as obviating the need for resolving the symbol grounding problem (Piantadosi & Hill, 2022). It also confirms the EP view that there are substantial informational patterns in the human‐structured environment, in a format appropriate to be taken advantage of by a suitably adapted learner. In this case, the textual outputs of language users allow at least a partial grasp by a learning system of the conceptualization of the world within a language community (provided a vast amount of data, parameters, and compute resources are available).

The success, as well as the dangers and vulnerabilities, of these systems can be attributed to their capacity to pick up and efficiently memorize regularities in their environment (i.e., the texts or other multimodal input being processed) with interpolation at inference time proving an effective strategy to deal with user interaction (Davies‐Barton et al., 2024), contrary to anybody's expectations until now. On the other hand, these models operate in stark contrast to what is observed by Beer (2023) for human cognitive systems: after training, these systems are mostly static in the sense that no further learning is pursued through online embodied interactions with the sociomaterial environment (although some temporary dynamic adaptation occurs online within the confines of a fixed context window). As Beer points out, in human agents, we cannot differentiate behavior from higher‐order capacities like action choice and (continuous) learning and adaptation (see also Dingemanse et al., 2023). Most current AI systems in contrast do exactly that since they neither learn in realistic interactive contexts with the potential to negotiate meaning while interacting with users nor possess embodiment that would allow learning by being coupled with the physical world. As a result, multiple failures and unreliability ensue since users expect human‐like interactions that are not currently achievable. Moreover, no substantial creativity and novelty are possible under the current regime even though, we believe, not in principle for AI (cf. Roli et al., 2022).

These kinds of failures of current AI architectures have led to a backlash of criticism seemingly motivated by the perspective of computationalism, Cartesian‐Newtonian dualism, and standard linguistic models. Critics of deep learning and current AI constantly point out that what is missing from such models is some notion of semantics and pragmatics which needs to be available independently from, and in addition to, the level of “forms” and syntax, which purportedly is the only linguistic level captured by such models (see, e.g., Bender & Koller, 2020; Bender et al., 2021). In congruence with Deacon's model of symbolic function, this would seem to diagnose the problem with LLMs as the fact that only a system of relations between forms is captured by these models which remain ungrounded through lack of indexical/iconic linking to objects in the world.

We believe that this is not the correct explanation for either the successes or the failures of current AI technologies and leads to paradoxical assumptions. Contrary to Deacon's aims, if we accept this explanation of LLMs’ performance, the “autonomy of syntax” hypothesis appears as a viable assumption since LLMs extract and manipulate syntactic patterns without prior semantic/pragmatic understanding. Under Deacon's model, the only explanation is that LLMs learn high‐level correlations (indexical relations) among tokens enabling them to simulate a symbolic capacity since there is neither a process of “ungrounding” nor grounding of such a symbolic level to any relations of reference or iconicity/indexicality with objects and their relations in the world. The claim of simulation is implausible. LLMs display conceptual abilities, however defective, in that high‐order semantic patterns are successfully picked up and deployed appropriately in tasks posed by users with the consequence that these models are becoming able to linguistically act in the world through their interaction with humans. Positing the need for separate grounding processes of linguistic forms is also refuted by multimodal models that generalize their conceptualization capacities across multiple sources of information with integrated effects despite conversion of all training data in unified forms of representation across modalities.

Another inconsistency that also arises is that Deacon's (1997) model takes the emergence of the symbolic level with its independent systemic relationships as explaining why no species other than humans possess communication systems that can be characterized as symbolic. Nevertheless, current AI technologies show that forms (sign vehicles) cannot be simply considered inert material structures that are required to stand in correspondence with some external reality in order to be grounded. Instead, as material/energetic processes, linguistic forms can bootstrap conceptual abilities through their potential for organizing interacting processes and involvement in the world. Any form of such involvement provides both human and nonhuman entities with agential powers when participating in integrated systems of distributed cognition with the basis of such powers appearing even at the most fundamental level in processes of material differentiations (Barad 2003, 2007). Given that processes interact and self‐organize with emergent results at various levels (Bickhard, 2021), the agency and intentionality of AI systems do not have to be taken as an all‐or‐nothing predetermined issue but as gradations of emergent capacities depending on purposes of use, degree of intervention, and the abilities of any other entities involved (see, also, Kockelman, 2011; Kiverstein et al., 2022).

### 3.4 Peircian distinctions versus landscapes of affordances

Consequently, we believe that the basic problem with Deacon's view is the second trichotomy in the Peircian interpretation: sign‐vehicle (*representamen*), *object*, and *interpretant* (suggestive of the syntax‐semantics‐pragmatics distinction, Morris, 1938). Within the framework of DS‐TTR, we have suggested that the standard distinction between syntax, semantics, and pragmatics is unwarranted and work has been done in implementing DS‐TTR within embodied agents (e.g., Hough et al., 2020) giving nonverbal actions the same status as verbal utterances with the aim of building grammars of human interaction rather than simply language use in the narrow sense. The practices and regularities that underpin linguistic capacities can be reformulated uniformly as learned anticipations of potential interactions (affordances) without foundational symbolic relationships with structure in the world (Bickhard, 2024). From our perspective, anticipatory models are fundamentally performative in that any agent must act to realize meaningful interactions. Actions, including linguistic actions, engender process organizations constituting what is standardly conceptualized as “form,” verbal or written, and such organizations are inherently functional without needing to define separate grounding mechanisms. The notion of *affordance* in its modern conceptions (e.g., Mangalam et al., 2024) eliminates the need for providing a relation between structure/form and meaning/function motivated by the requirement of enabling reference to an object (see also Rączaszek‐Leonardi, 2021). What is directly perceived primarily by language users are the functions of linguistic actions rather than abstracted forms. The latter can become available in their own right as further differentiations of the coordinative structures underpinning human interactions but they do not constitute the primary stimulus that needs to be enriched or endowed with function/meaning as a separate stage of interpretation. Under the Peircian framework, at most, the notion of the interpretant, if appropriately conceived as constituting selection among the anticipatory dynamics, should be sufficient. In such a dyadic view, sign and interpretant, “semiotic” relations can be characterized as affordances all the way. Given the material/energetic notion of linguistic constraints emphasized by Rączaszek‐Leonardi in previous work (see also Rączaszek‐Leonardi et al., 2022), this is compatible with her account while situating it within new materialist positions in philosophy and theory of science describing processes that enact “agential cuts” in the production/performance of reality (Barad 2003, 2007) as opposed to establishing the necessity of an ontologically irreducible structure‐function complementarity.

## 4 Conclusion

Beer and Rączaszek‐Leonardi have opened a discussion of what is further needed to enhance the reach of the dynamical approaches to cognition. Focusing on issues concerning the nature of language and the development of language technology, we have attempted, in this brief contribution, to place their proposals in a larger philosophical framework that suggests lines of inquiry that we believe will yield fruitful outcomes.
